# Retracted: A Comparison between Three-Dimensional Visualization Guided Hepatectomy and Ultrasonography Guided Radiofrequency Ablation in the Treatment of Small Hepatocellular Carcinoma within the Milan Criteria

**DOI:** 10.1155/2017/8014852

**Published:** 2017-06-07

**Authors:** BioMed Research International

At the request of the authors, the article titled “A Comparison between Three-Dimensional Visualization Guided Hepatectomy and Ultrasonography Guided Radiofrequency Ablation in the Treatment of Small Hepatocellular Carcinoma within the Milan Criteria” [1] has been retracted. The article was found to contain substantial errors, as detailed below, which mean the data do not support the conclusions. The authors apologize for these errors.

(1) Nine patients were wrongly classified. Five patients in the surgery group with mixed hepatocellular carcinoma, cholangiocarcinoma in the postoperative pathological analysis, were wrongly recorded as having hepatocellular carcinoma based on intraoperative frozen section examination. Four patients in the radiofrequency ablation group had severe dysplasia rather than hepatocellular carcinoma according to histopathology in the follow-up period.

When these patients are excluded, liver function is a risk factor for survival (hazard ratio = 1.8, *p* = 0.015), but this parameter was wrongly excluded from the risk factors in the published article.

(2) There was high multicollinearity between liver function of Child-Pugh Class and Tbil, Alb, and PT (eigenvalue = 0.07≈0, condition index = 20.484 > 10; Table 1). Due to this, the results may change unpredictably in response to small changes in the model or the data [2]. To overcome this issue, we need a larger sample size and improved statistical methodologies [3].

## Figures and Tables

**Table 1:** Results of the collinearity diagnostics.

Collinearity diagnostics^a^							
Model	Dimension	Eigenvalue	Condition index	Variance proportions			
				(Constant)	Tbil	ALB	PT
1	1	2.972	1.000	0.00	0.02	0.00	0.02
	2	0.845	1.876	0.00	0.01	0.00	0.97
	3	0.177	4.103	0.01	0.91	0.02	0.00
	4	0.007	20.484	0.99	0.06	0.98	0.01

^a^Dependent variable: Child-Pugh.
